# Cultural Adaptation of a Validated Intervention for Chinese and Korean American Dementia Caregivers

**DOI:** 10.1093/geroni/igaf122.419

**Published:** 2025-12-31

**Authors:** Eunjung Ko, Bei Wu, Yaolin Pei, Xiang Qi, I tek Leong, Weiyu Mao, Jing Wang, Mary Mittelman

**Affiliations:** New York University, New York, New York, United States; NYU Shanghai, Shanghai, China (People’s Republic); The University of Texas at Austin, Austin, Texas, United States; New York University, New York, New York, United States; New York University, New York, New York, United States; University of Nevada, Reno, Reno, Nevada, United States; University of New Hampshire, Durham, New Hampshire, United States; New York University Grossman School of Medicine, New York, New York, United States

## Abstract

With the growing diversity of the U.S. population, culturally adapted interventions are essential in dementia caregiving. However, approaches tailored for East Asian cultures remain limited. Based on our ongoing randomized controlled trial of the, “New York University Caregiver Intervention—Enhanced Support (NYUCI-ES),” we identified adaptation strategies for Chinese and Korean cultures and categorized them into five elements: content, context, relationship fidelity to core elements, engagement, and cultural competence. Content adaptations included translating materials into multiple languages, and incorporating culturally relevant terminology, questionnaires, community resources, and social media support groups. For context, we delivered the counseling sessions either in person or online, arranged culturally matched staff, trained staff to provide culturally tailored counseling, and established strong partnerships with Korean and Chinese organizations. Fidelity to the original intervention was maintained through the original developers’ active involvement in regular meetings that also include experts in East Asian culture, and the detailed explanation about the NYUCI-ES in participants’ languages. Engagement strategies included step-by-step efforts at involving prospective participants, rapport through explanation of staff roles, multiple communications, and participant compensation. For cultural competence, we used a culturally tailored counseling approach, respecting cultural beliefs on counseling, illness, and family, used euphemistic language about dementia, and provided culturally appealing refreshments. Within the NYUCI’s structured framework, we adapted content to East Asian culture. These strategies address research gaps and can guide future cultural adaptations of evidence-based interventions to the preferences and needs of members of East Asian communities to improve their physical and emotional health.

